# The Outcome of Double Antibiotic, Triple Antibiotic, and Modified Triple Antibiotic Pastes as an Intracanal Medicament in Regenerative Endodontics: A Comparative Study

**DOI:** 10.7759/cureus.110018

**Published:** 2026-06-01

**Authors:** Takhellambam P Devi, Shamurailatpam Priyadarshini, Kangjam G Singh, Deepak B Suryakanth, Jenny Atom, Sagolsem Chandarani

**Affiliations:** 1 Department of Conservative Dentistry and Endodontics, Dental College, Regional Institute of Medical Sciences (RIMS), Imphal, IND

**Keywords:** double antibiotic paste, intracanal medicament, modified triple antibiotic paste, regenerative endodontics, stem cells, success, triple antibiotic paste

## Abstract

Background

Antibiotic paste plays a vital role in the success of regenerative endodontics. Triple antibiotic paste (TAP) and double antibiotic paste (DAP) are commonly used as intracanal medicaments, with tooth discoloration being a main concern for TAP. Clindamycin is a broad-spectrum antibiotic and does not cause visible tooth discoloration. This study was conducted to evaluate and compare the clinical and radiographic outcomes of regenerative endodontics using DAP, TAP, and modified triple antibiotic paste (mTAP) as intracanal medicaments.

Methodology

A total of 51 non-vital immature permanent teeth from 51 patients aged 12-25 years were included in this comparative study. The teeth were randomly allocated into the following three groups (n = 17) based on the intracanal medicament used: Group I: DAP was used as an intracanal medicament; Group II: TAP was used as an intracanal medicament; and Group III: mTAP, in which the minocycline of TAP was replaced by clindamycin, was used as an intracanal medicament. After completion of regenerative endodontic treatment, the teeth were followed clinically and radiographically at 12 and 24 months. The chi-square and Fisher’s exact tests were used to compare the outcome of regenerative endodontics among the three groups.

Results

At the 12-month follow-up, the success rates for regenerative endodontics were 87.50%, 88.24%, and 82.35% for the DAP, TAP, and mTAP groups, respectively, and there were no statistically significant differences (p = 0.999). At the 24-month follow-up, the success rates for regenerative endodontics were 68.75%, 81.25%, and 73.33% for the DAP, TAP, and mTAP groups, respectively, and there were no statistically significant differences (p = 0.775).

Conclusions

Regenerative endodontics using DAP, TAP, and mTAP as intracanal medicaments have high success rates with no statistically significant differences among the three. mTAP, in which the minocycline of TAP is replaced by clindamycin, can also be used as an intracanal medicament in regenerative endodontics.

## Introduction

Endodontic therapy in immature permanent teeth poses a great challenge due to open apices and thin dentinal walls. Apexification and regenerative endodontics are treatment options for preserving teeth in such cases. Regenerative therapy offers hope for transforming a non-vital tooth into a vital tooth once again [1]. Regenerative endodontics is a biological procedure designed to replace damaged structures, including dentin, root structures, and cells of the pulp-dentin complex [1,2]. This procedure has proven to produce a favorable biological outcome compared to the conventional apexification technique, which fails to promote continued root development, leaving teeth more susceptible to fracture [3].

Proper disinfection of the root canal space and maintaining stem cell viability, as well as promoting their proliferation and differentiation, are essential for the success of regenerative endodontics [4]. No or minimal instrumentation is recommended due to the presence of thin dentinal walls. Hence, disinfection in regenerative endodontics mainly involves the use of intracanal irrigants and the placement of intracanal medicaments, especially antibiotics, for several weeks [4,5]. Infections of the root canal system are polymicrobial in nature; therefore, a combination of antibiotics is required. Triple antibiotic paste (TAP) comprises metronidazole, ciprofloxacin, and minocycline, and is commonly used as an intracanal medicament in regenerative endodontics, with promising results [6]. Metronidazole is a nitroimidazole compound effective against anaerobic cocci as well as Gram-positive and Gram-negative bacilli. It kills bacteria by permeating their cell membranes and then binding to DNA, causing disruption of its helical structure. Ciprofloxacin is a second-generation fluoroquinolone with a broad spectrum of activity against Gram-negative microorganisms. It inhibits DNA gyrase in bacterial nuclei, degrades DNA by exonucleases, and results in a bactericidal effect. Minocycline is a bacteriostatic group of broad-spectrum antibiotics effective against both Gram-positive and Gram-negative microorganisms. It enters bacterial cells by passive diffusion across the outer membrane, followed by active transport through the inner membrane, reaching the surfaces of ribosomes and inhibiting protein synthesis. In addition to its antimicrobial activity, it inhibits collagenases and clastic cells, resulting in anti-resorptive properties [6-8].

TAP effectively disinfects necrotic infected pulp and creates an environment conducive to vital tissue regeneration [7]. However, one of the main concerns with TAP is tooth discoloration, as the minocycline component of TAP binds to calcium ions in the tooth, forming an insoluble chelate [9]. As a result, double antibiotic paste (DAP), which omits minocycline, and modified triple antibiotic paste (mTAP) with alternatives to minocycline, such as clindamycin, amoxycillin, or cefaclor, have been considered [10,11]. Clindamycin, a bacteriostatic lincosamide, is effective against a broad spectrum of endodontic bacteria (Gram-positive aerobes and most anaerobic bacteria), making it a clinically viable alternative to minocycline [12]. Moreover, it exhibits an antibiofilm effect comparable to TAP [13].

To our knowledge, no previous study has evaluated and compared the clinical and radiographic outcomes of regenerative endodontics using DAP, TAP, and mTAP (minocycline of the TAP replaced by clindamycin) as intracanal medicaments. Hence, this study aimed to evaluate and compare the clinical and radiographic outcomes of regenerative endodontics using DAP, TAP, and mTAP as intracanal medicaments. The proposed null hypothesis was that there would be no statistically significant differences in the clinical and radiographic outcomes of regenerative endodontics using DAP, TAP, and mTAP as intracanal medicaments.

## Materials and methods

**Figure 1:**
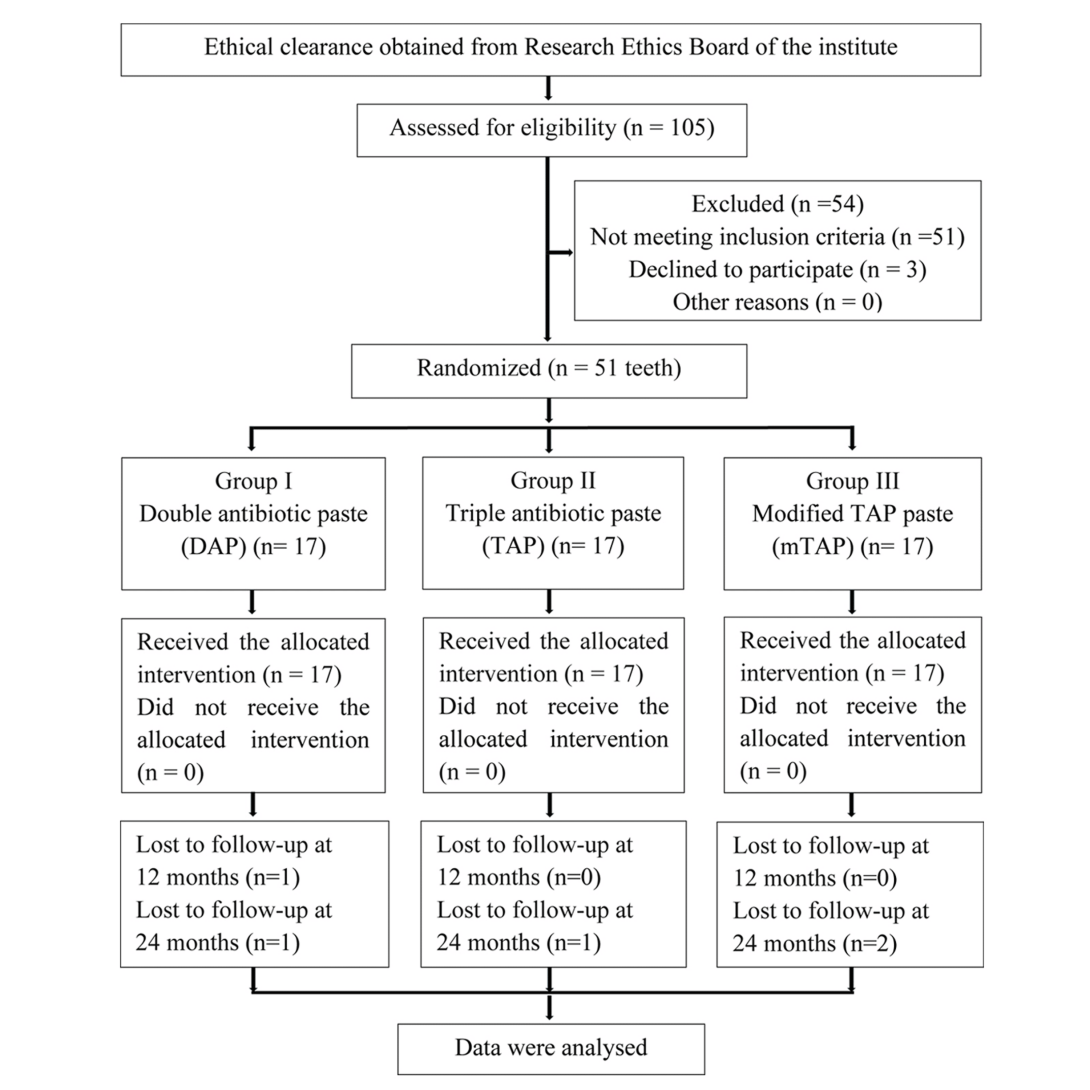
Consolidated Standards of Reporting Trials (CONSORT) flow diagram.

This clinical study was approved by the Research Ethics Board of the institute (approval number: A/206/REB/Prop(Faculty)137/64/2020 dated 16-12-2020). The study was conducted in accordance with the Consolidated Standards of Reporting Trials (CONSORT) guidelines (Figure 1).

Patients were enrolled between January 2021 and January 2023 from outpatient department referrals to the Department of Conservative Dentistry and Endodontics. The overall study period, including follow-up, was from January 2021 to March 2025. Systematically healthy patients in the age group of 12-25 years with non-vital immature permanent teeth in Cvek’s stages 1, 2, and 3 of root formation were included. Patients with immunocompromised conditions, primary teeth, teeth in Cvek’s stages 4 and 5 of root formation, periodontally compromised teeth, traumatized teeth, root fracture, and patients unwilling to participate were excluded.

A convenience sampling technique was used. The sample size included all eligible patients who presented to the department during the defined study period. In total, 51 non-vital immature permanent teeth from 51 patients were recruited in accordance with the inclusion criteria. Written informed consent was obtained from the patient or a parent/guardian if the patient was under 18 years of age. Computer-generated block randomization with a block size of 3 was performed to randomly allocate 51 teeth into three groups (n = 17 each). The allocations were placed in sequentially numbered sealed envelopes. The three groups were classified based on the intracanal medicament.

Treatment procedure

The treatments were performed by a single operator in the Department of Conservative Dentistry and Endodontics. The teeth were anesthetized with 2% lidocaine hydrochloride with epinephrine 1:80,000. A rubber dam was applied, and the access cavity was prepared with an endo access bur. Working length was determined using a paper point technique and confirmed radiographically with the use of a K-file. Minimal instrumentation of the canal was performed. The canals were copiously irrigated with 1.5% sodium hypochlorite (NaOCl) (20 mL/canal, five minutes) followed by normal saline (20 mL/canal, five minutes) using a side‑vented needle positioned 1 mm from the working length. The canals were dried with paper points, and intracanal medicament was placed below the cemento-enamel junction (CEJ). Based on the intracanal medicaments, the teeth were divided into three groups of 17 teeth each. In Group I (DAP group), 1 mg/mL DAP, composed of ciprofloxacin and metronidazole in a 1:1 ratio in distilled water, was used. In Group II (TAP group), 1 mg/mL TAP, composed of ciprofloxacin, metronidazole, and minocycline in a 1:1:1 ratio in distilled water, was used. In Group III (mTAP group), 1 mg/mL mTAP, composed of ciprofloxacin, metronidazole, and clindamycin in a 1:1:1 ratio in distilled water, was used.

**Figure 2:**
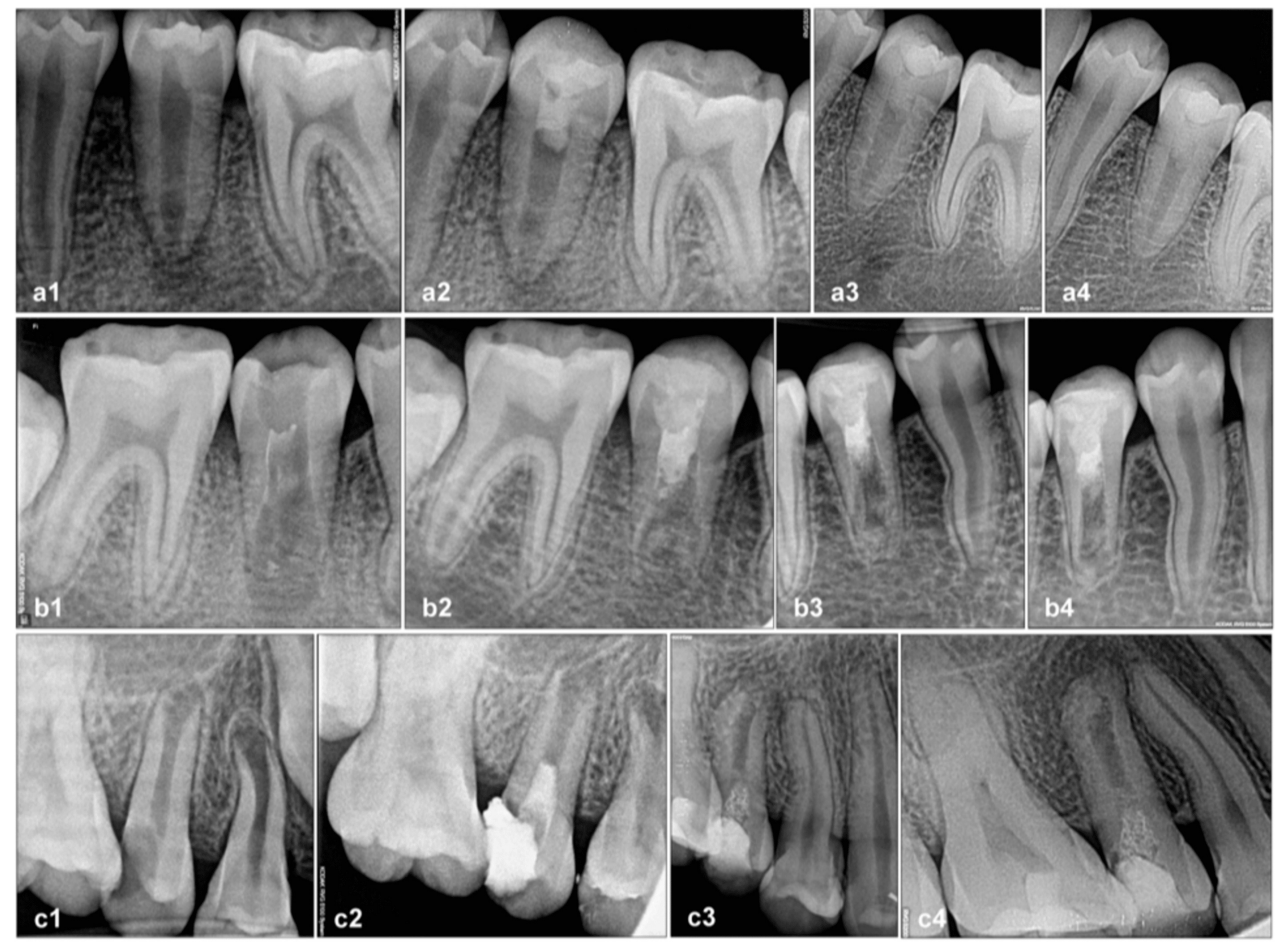
Representative radiographs of Group I (Case a), Group II (Case b), and Group III (Case c). (1) Preoperative radiograph; (2) immediate postoperative radiograph; (3) 12-month follow-up; and (4) 24-month follow-up.

The access cavity was sealed with a temporary restorative material (IRM, Dentsply Sirona). The patients were recalled at two weeks, and if symptomatic, the previous irrigation protocol and placement of intracanal medicament were repeated. If the tooth was asymptomatic, it was anesthetized with 3% mepivacaine without epinephrine. Under rubber dam isolation, the canals were copiously irrigated with 17% EDTA (20 mL/canal, five minutes). The canals were dried with paper points, and bleeding was induced by over-instrumenting using a #25 K-file introduced 2 mm beyond the apical foramen, so that the entire canal was filled with blood up to the level of the CEJ. A cotton pellet moistened with normal saline was placed for 10 minutes to allow blood clot formation. Over the blood clot, 3 mm of ProRoot MTA (Dentsply, Tulsa, OK, USA) was placed, followed by a resin-modified glass ionomer and a composite restorative material. A postoperative intraoral periapical radiograph was taken. The cases were followed up clinically and radiographically at 12 and 24 months (Figure 2).

The outcomes were assessed clinically and radiographically by an expert endodontist blinded to the study according to the American Association of Endodontists’ clinical considerations for a regenerative procedure [11]. The outcome of regenerative endodontic procedures was assessed by the extent to which the primary, secondary, and tertiary outcome goals were achieved. The primary goal was the elimination of symptoms and evidence of bony healing [11]. Success was defined as the absence of clinical signs and symptoms and radiographic evidence of bony healing. The survival and failure were also reported. Survival was defined as the tooth retained without clinical signs or symptoms, regardless of radiographic signs of disease. Failure was defined as the presence of clinical signs or symptoms and/or the persistence of or an increase in periapical radiolucency, or the tooth was endodontically treated or was extracted [14].

The secondary goal was increased root wall thickness and/or an increased root length (desirable, but perhaps not essential). The tertiary goal was a positive response to vitality testing (which, if achieved, could indicate a more organized vital pulp tissue) [11]. The pulp sensibility test was performed by cold test and electric pulp test (EPT) at 12-month and 24-month follow-ups. The root length was measured from the CEJ to the midpoint of the root apex. The root wall thickness was measured at the apical third of the preoperative radiograph and at the same length for the follow-up radiographs. The root wall thickness was calculated by subtracting the width of the root canal from the width of the root [15].

Statistical analysis

Statistical analysis was performed using SPSS software for Windows version 26.0 (IBM Corp., Armonk, NY, USA). One-way analysis of variance (ANOVA) was used to compare the mean ages (years) of patients among groups. Chi-square and Fisher’s exact tests were used to compare the proportions among groups. A P-value <0.05 was considered statistically significant.

## Results

Of the 51 patients treated, 26 were male, and 25 were female. The treated teeth comprised anterior, premolar, and first molar, with the majority being premolars. There were no statistically significant differences in age (p = 0.189), gender (p = 0.361), tooth types (p = 0.424), or preoperative symptoms (p = 0.577) among the DAP, TAP, and mTAP groups (Table 1). The causes of non-vitality of teeth were dental caries and broken dens evaginatus.

**Table 1:** Preoperative data of participants. P-value <0.05 was considered statistically significant. DAP: double antibiotic paste; TAP: triple antibiotic paste; mTAP: modified triple antibiotic paste; SD: standard deviation; F: one-way analysis of variance; X²: chi-square test; p: Fisher’s exact test

Variables	Categories	DAP group (n = 17)	TAP group (n = 17)	mTAP group (n = 17)	P-value	Test statistics
Age (years)	Mean ± SD	14.7 ± 2.14	15.5 ± 2.67	16.2 ± 2.34	0.189	F = 1.724
Gender	Male	8	7	11	0.361	X^2 ^= 2.040
	Female	9	10	6		
Tooth type	Anterior teeth	3	2	1	0.424	p = 3.407
	Premolar teeth	12	14	16		
	Molar teeth	2	1	0		
Symptoms	Symptomatic	15	12	13	0.577	p = 1.648
	Asymptomatic	2	5	4		

At the 12-month follow-up, one patient was lost to follow-up in the DAP group. The success rates for regenerative endodontics were 87.50%, 88.24%, and 82.35% for the DAP, TAP, and mTAP groups, respectively, with no statistically significant differences (p = 0.999). Root development (thickening or lengthening or decrease in apical diameter) were 62.50%, 58.82%, and 52.94% for the DAP, TAP, and mTAP groups, respectively, with no statistically significant differences (p = 0.854). Positive responses to pulp sensibility test were 6.25%, 5.88%, and 0% for the DAP, TAP, and mTAP groups, respectively, with no statistically significant differences (p = 0.764) (Table 2). Teeth without any clinical signs or symptoms were followed further.

**Table 2:** Intragroup comparison of outcomes at the 12-month follow-up. P-value <0.05 was considered statistically significant. DAP: double antibiotic paste; TAP: triple antibiotic paste; mTAP: modified triple antibiotic paste; EPT: electric pulp test; X²: chi-square test; p: Fisher’s exact test

Outcomes	DAP group (n = 16)	TAP group (n = 17)	mTAP group (n = 17)	P-value	Test statistics
Survival (elimination of symptoms)	14 (87.50%)	16 (94.12%)	15 (88.24%)	0.999	X^2 ^= 0.393
Success (elimination of symptoms and evidence of bony healing)	14 (87.50%)	15 (88.24%)	14 (82.35%)	0.999	X^2 ^= 0.426
Failure	2 (12.50%)	2 (11.76%)	3 (17.65%)	0.999	p = 0.426
Root development					
Thickening	6 (37.50%)	6 (35.29%)	5 (29.41%)	0.878	X^2 ^= 0.260
Lengthening	2 (12.50%)	2 (11.76%)	1 (5.88%)	0.860	p = 0.679
Decrease in apical diameter	6 (37.50%)	7 (41.18%)	8 (47.06%)	0.854	X^2 ^= 0.316
Thickening or lengthening or a decrease in apical diameter	10 (62.50%)	10 (58.82%)	9 (52.94%)	0.854	X^2 ^= 0.316
No development	6 (37.50%)	7 (41.18%)	8 (47.06%)	0.854	X^2 ^= 0.316
Pulp sensibility (either EPT or cold test)					
Positive response	1 (6.25%)	1 (5.88%)	0 (0%)	0.764	p = 1.326
Negative response	15 (93.75%)	16 (94.11%)	17 (100%)		
Crown discoloration					
Present	4 (25.00%)	5 (29.41%)	3 (17.65%)	0.779	p = 0.727
Absent	12 (75.00%)	12 (70.59%)	14 (82.35%)		

At the 24-month follow-up, one patient was lost to follow-up in the DAP and TAP groups, and two patients in the mTAP group. The success rates for regenerative endodontics were 68.75%, 81.25%, and 73.33% for the DAP, TAP, and mTAP groups, respectively, with no statistically significant differences (p = 0.775). Root development (thickening or lengthening or decrease in apical diameter) were 68.75%, 75.00%, and 66.67% for the DAP, TAP, and mTAP groups, respectively, with no statistically significant differences (p = 0.924). Positive responses to pulp sensibility test were 6.25%, 12.50%, and 0% for the DAP, TAP, and mTAP groups, respectively, with no statistically significant differences (p = 0.763) (Table 3).

**Table 3:** Intragroup comparison of outcomes at the 24-month follow-up. P-value <0.05 was considered statistically significant. DAP: double antibiotic paste; TAP: triple antibiotic paste; mTAP: modified triple antibiotic paste; EPT: electric pulp test; X²: chi-square test; p: Fisher’s exact test

Outcomes	DAP group (n = 16)	TAP group (n = 16)	mTAP group (n = 15)	P-value	Test statistics
Survival (elimination of symptoms)	11 (68.75%)	13 (81.25%)	11 (73.33%)	0.775	X^2 ^= 0.742
Success (elimination of symptoms and evidence of bony healing)	11 (68.75%)	13 (81.25%)	11 (73.33%)	0.775	X^2 ^= 0.742
Failure	5 (31.25%)	3 (18.75%)	4 (26.67%)	0.775	p = 0.742
Root development					
Thickening	7 (43.75%)	8 (50.00%)	6 (40.00%)	0.851	X^2 ^= 0.322
Lengthening	2 (12.50%)	2 (12.50%)	1 (6.67%)	0.999	p = 0.548
Decrease in apical diameter	9 (56.25%)	11 (68.75%)	10 (66.67%)	0.734	X^2 ^= 0.618
Thickening or lengthening or a decrease in apical diameter	11 (68.75%)	12 (75.00%)	10 (66.67%)	0.924	X^2 ^= 0.373
No development	5 (31.25%)	4 (25.00%)	5 (33.33%)	0.924	p = 0.373
Pulp sensibility (either EPT or cold test)					
Positive response	1 (6.25%)	2 (12.50%)	0 (0%)	0.763	p = 1.823
Negative response	15 (93.75%)	14 (87.50%)	15 (100%)		
Crown discoloration					
Present	6 (37.50%)	8 (50.00%)	5 (33.33%)	0.613	X^2 ^= 0.979
Absent	10 (62.50%)	8 (50.00%)	10 (66.67%)		

## Discussion

Dental caries, dental trauma, or anatomical anomalies can lead to pulp necrosis and pose clinical challenges in treating immature permanent teeth. Moreover, these teeth are susceptible to fracture and tooth loss due to incomplete root development [16]. Regenerative endodontics is a biologically based treatment that is being recognized as the first treatment of choice for immature permanent teeth with pulp necrosis. The clinical considerations for regenerative endodontics include disinfection of the root canal, placement of a scaffold within the root canal, often involving laceration of the periapical tissue to induce a blood clot and initiate stem cell activity, and an adequate coronal seal to prevent reinfection [17]. Stem cells contribute to continued root maturation in immature teeth and to pulp/dentin regeneration [18].

A concentration of 1.5% NaOCl was used in the study, as a lower concentration is equally effective and minimizes cytotoxicity to stem cells in the apical tissues. A higher concentration of NaOCl significantly decreases the survival of stem cells of the apical papilla (SCAP) [19]. Further, 17% EDTA was used because it results in the release and quantification of growth factors from dentin, increases SCAP survival expression, and partially reverses the deleterious effects of NaOCl [19,20]. Antibiotic paste at a concentration of 1 mg/mL was used, as this concentration provides a balance for stem cell survival and antimicrobial effect [5].

The success rate of regenerative endodontics ranges from 50% to 98% [21]. This wide range of success can be due to variations in the causes of non-vitality, age of patients, treatment protocols, and outcome measurements [22]. The results of the present study showed that the success rates for regenerative endodontics using DAP, TAP, and mTAP as intracanal medicaments were statistically non-significant. The literature also reports high success rates for regenerative endodontics using DAP and TAP, with no significant difference [23]. A previous study reported a high success rate for regenerative endodontics using mTAP [24]. The majority of failed cases in the three groups were reported with painful conditions before the recall period. These teeth were treated with an apexification procedure using MTA.

Regarding root development, a decrease in apical diameter was observed in most teeth, followed by root thickening and root lengthening, with the least in all three groups. A similar pattern was observed for TAP in a previous study [14].

The results of the present study showed that a very few teeth responded to the pulp sensibility test. This may be due to capping with MTA at the CEJ level. Moreover, the tissues formed were more reparative than regenerative [25]. The literature shows a wide range of responses to pulp sensibility tests after regenerative endodontics, which may be attributed to unreliable subjective responses from young patients [14]. The results of the present study showed that tooth discoloration increases with longer follow-up. This can be attributed to the use of MTA as a capping material in all three groups. The number of teeth discoloration was slightly more in the TAP group than in the other groups, which could be due to the minocycline content of TAP.

The limitation of the present study is the use of a two-dimensional radiograph to assess the outcome of regenerative endodontics.

## Conclusions

Within the limitations of this study, mTAP demonstrated clinical and radiographic outcomes comparable to DAP and TAP in regenerative endodontics. Further multicenter randomized trials with larger sample sizes are needed to validate these findings. Regarding root development, a decrease in apical diameter occurred the most, followed by root thickening and root lengthening, with the least in all three groups, with no statistically significant differences among the three. Moreover, positive responses to the pulp sensibility test were observed in a few teeth in the DAP, TAP, and mTAP groups, with no statistically significant differences among the three. Based on the findings of the present study, mTAP can be considered an intracanal medicament in regenerative endodontics.
